# Elicitor-Induced Defense Responses in *Solanum lycopersicum* against *Ralstonia solanacearum*

**DOI:** 10.1155/2013/561056

**Published:** 2013-09-25

**Authors:** Sudhamoy Mandal, Itishree Kar, Arup K. Mukherjee, Priyambada Acharya

**Affiliations:** ^1^Plant Pathology Laboratory, Central Horticultural Experiment Station (ICAR), Aiginia, Bhubaneswar 751019, India; ^2^Divisin of Crop Protection, Central Rice Research Institute (ICAR), Bidyadharpur, Cuttack 753006, India

## Abstract

We investigated on important parameters of induced resistance in hydroponic tomato (*Solanum lycopersicum*) against *Ralstonia solanacearum* using the elicitors chitosan (CHT), salicylic acid (SA), and jasmonic acid (JA). The increase in total phenolic content of roots by the elicitors was significantly higher than control. Most pronounced increase in lignin synthesis was triggered by SA followed by CHT. At 24 h post-elicitation (hpe), the activity of phenylalanine ammonia lyase was 4.5 times higher than control elicited by CHT. The peroxidase activity was about 86 nkat/mg protein at 24 hpe in case of SA and 78 nkat/mg protein in case of CHT. The activity of polyphenol oxidase increased several folds by the elicitors. Cinnamyl alcohol dehydrogenase activity increased to the maximum at 48 hpe under the influence of CHT. The results indicate that the elicitors SA and CHT induced effective defense responses in tomato plants against *R. solanacearum*. This was evident from reduced vascular browning and wilting symptoms of tomato plants treated with SA and CHT and challenged subsequently with *R. solanacearum*. This reduced disease incidence in tomato by SA and CHT may be a result of cell wall strengthening through deposition of lignin and the coincident induction of defense enzymes.

## 1. Introduction

*Ralstonia solanacearum* (causal organism of bacterial wilt) is a major plant pathogen that attacks important crops and other plants over a broad geographical range. The extensive genetic diversity of strains responsible for the various bacterial wilt diseases of plants has led to the concept of a *Ralstonia solanacearum *species complex [1]. One of the main constraints of tomato (*Solanum lycopersicum *L.) cultivation today is losses caused by bacterial wilt [2]. Bacterial wilt on tomato crops appears as a sudden wilt. Infected young plant dies rapidly. Older plant first shows wilting of the youngest leaves, or one sided wilting and stunting, and then finally the plant wilts permanently. The vascular tissue of stems and roots turns brown and in cross-section they ooze whitish bacterial exudates. 

Plants deploy a battery of mechanisms to defend themselves against pathogen infection. They have evolved complex defense strategies that include both constitutive and pathogen-induced components [3]. The constitutive defenses of plants include structural barriers, such as plant cell wall as well as inhibitory compounds including phenolics [4]. Soluble and cell wall-bound phenolic compounds accumulate in plant tissues challenged by fungal pathogens [5]. These phenolics play an important role in the resistance of plants to pathogen attack as they belong to the antimicrobial defense arsenal [6]. Esterification of phenolics to cell wall materials is a common theme in the expression of plant resistance [7]. Lignin is the main structural component of secondarily thickened plant cell walls. In the last step of monolignol biosynthesis, coniferaldehyde is converted into its corresponding alcohol by cinnamyl alcohol dehydrogenase (CAD) in a NADPH-dependent reaction [8].

Enhancing host resistance using elicitors that addresses environmental concern can be an effective disease control strategy. Inducible plant defenses are triggered by the perception of a pathogen or elicitors. Perception of elicitors takes place in receptors located either at the cell surface or inside the cell [9]. This recognition of elicitors triggers overlapping signaling responses in the plant [10]. Recognition of the elicitor induces several early responses in plants. Salicylic acid (SA) has been shown to be an important signalling molecule involved in defense responses to pathogen attack in many plant-pathogen interactions [11]. We demonstrated earlier that exogenous application of 200 mM salicylic acid through root feeding and foliar spray could induce resistance against *Fusarium oxysporum* f. sp. *lycopersici* in tomato [12]. Chitosan (CHT), a deacetylated chitin derivative and a natural, nontoxic homopolymer, behaves like a general elicitor inducing a nonhost resistance and priming a systemic acquired immunity [13]. Phenotypic effects in the tomato plant following induced resistance affected by CHT were manifested in bacterial reduction in plant tissue and reduced wilt incidence [14]. Jasmonic-acid- (JA-) dependent defense responses were suggested to contribute to basal resistance in tomato against different pathogens and to play a role in regulation of systemic defense responses after pathogen attack [15].

Phenylalanine ammonia lyase (PAL) is a key enzyme of phenylpropanoid metabolism in plants. PAL is an important enzyme in the biosynthesis of phenolic compounds in tomato [16]. The enhanced resistance of acibenzolar-S-methyl- (ASM-) treated tomato plants against *Clavibacter michiganensis *ssp. *michiganensis* was associated with significant increases in peroxidase (POD) activities [17]. Polyphenol oxidase (PPO) is important in defense against pathogens through its role in oxidation of phenolic compounds into antimicrobial quinines [18]. CAD activity is an indicator of lignin biosynthesis because of its specific role at the end of the monolignol biosynthetic pathway [19].

We investigated on important parameters of induced resistance in hydroponically grown tomato against* Ralstonia solanacearum* (*Ralsol*) using three elicitors *namely*, chitosan (CHT), salicylic acid (SA), and jasmonic acid (JA).

## 2. Materials and Methods

### 2.1. Plant Material

*Solanum lycopersicum *L. cv. Arka Meghali was used in the research project. The seeds of the tomato variety Arka Meghali were obtained from last season's crop. This variety gives high yield, but it has been found susceptible to bacterial wilt. 

The plants were grown in a fertile and irrigated plot in open ambient climate as well as in hydroponics culture according to experimental requirements. 

### 2.2. The Pathogen

A highly virulent strain of *Ralsol* (phylotype 1, biovar 3) was cultured by streaking a single colony on *Pseudomonas solanacearum* medium (HiMedia, India) and incubating at 30°C for 48 h. The bacterial strain was isolated from a diseased eggplant and its biovar was determined based on its ability to utilize disaccharides (lactose, maltose, and cellobiose) and hexose alcohols (mannitol, sorbitol, and dulcitol). 

### 2.3. Establishment of Hydroponics Culture of Tomato Plants

 Tomato seeds were sown in soil medium and grown to the 4-week stage in open ambient climate. Plants were removed from the soil, and the growing soil medium was washed away from the roots with a gentle stream of water. The roots were then placed into hydroponics vessels (250 mL conical flasks) containing 300 mL Knop's nutrient medium. The hydroponics vessels were painted black on the exterior to decrease light penetration in order to prevent algal growth in the liquid medium. Hydroponically grown plants were maintained under a 14 h photoperiod regime at 24 ± 1°C in a growth chamber.

### 2.4. Elicitors and Treatment of Hydroponically Grown Tomato 

#### 2.4.1. Elicitor Preparation

Three elicitors used in the elicitation experiment were chitosan (CHT), salicylic acid (SA), and jasmonic acid (JA). CHT was prepared essentially as described by Villegus and Brodelius [20]. SA and JA were dissolved in sterile distilled water (with 10% methanol) at 0.01% concentration.

#### 2.4.2. Treatment of Tomato Plants with Elicitors

After the plants were established in the hydroponics culture, elicitors were added to the media. CHT was added at final concentration of 0.1%, and SA and JA were added at a final concentration of 0.01%. Nutrient medium containing the elicitors was exchanged daily for 7 days to ensure a continuous supply of nutrients and required concentration of the elicitors.

#### 2.4.3. Pathogen Inoculation of Tomato Plants

Bacterial inoculum was prepared from agar plates by flooding with sterile distilled water, and an optical density of 0.05 at 600 nm, corresponding to approximately 7.0 × 10^6^ colony-forming units per milliliter (CFU/mL), was adjusted. Tomato plants treated with elicitors as well as control plants were inoculated with addition of 2.5 mL of bacterial inoculum (2.5 mL sterile distilled water in control) in the hydroponics media (approx. 300 mL) after two days (i.e., 48 h) of last elicitor application. As additional control, noninoculated tomato plants were used. Plants were maintained in a growth chamber under the same conditions as described elsewhere. Both tap roots and fibrous roots of the plants were harvested on a time course to perform analyses.

### 2.5. Time Course Studies on Defense Responses

#### 2.5.1. Estimation of Total Phenolics from Roots of Tomato

The total phenolic content was determined as described by Mandal et al. [21] using Folin-Ciocalteau reagent. The reaction mixture contained 100 *μ*L of methanolic extract of eggplant root tissues and 200 *μ*L sterile distilled water with 500 *μ*L of Folin-Ciocalteau reagent. After 5 min, 800 *μ*L of 20% sodium carbonate was added, and after 1 h of incubation, the absorbance was measured at 254 nm in a BioMate 3 spectrophotometer (Thermo Spectronic, USA). Standard curve was prepared with *p*-hydroxybenzoic acid in 50% (v/v) methanol. The total phenolic content was expressed as micrograms of *p*-hydroxybenzoic acid equivalent/g FW of tomato root tissues.

#### 2.5.2. Determination of Lignin in Elicited Tomato Roots

Lignin was extracted according to the method of Bruce and West [22] with slight modifications. Briefly, root segments (elicited and control) were homogenized in 80% methanol. The homogenate was filtered through Whatman no. 4 filter paper and rinsed with methanol. Then the residue was dried at 60°C for 24 h. The dried alcohol insoluble residue (AIR) was used for the lignin determination. To 50 mg of AIR in glass screw-cap vials were added 5 mL HCl (2 N) and 0.5 mL thioglycolic acid (TGA), and the mixture was placed in boiling water for 4 h. The mixture was then centrifuged at 20 000 ×g for 15 min and the pellet was washed with 5 mL deionised water. The resulting pellet was suspended in 5 mL of 0.5 N NaOH, shaken at 25°C for 2 h, and then centrifuged at 20 000 ×g for 15 min. Concentrated HCl (1 mL) was added to the supernatant and the lignin-thioglycolic acid was allowed to precipitate at 4°C for 4 h. After centrifugation at 10 000 ×g for 10 min, the orange-brown pellet was dissolved in 10 mL of 0.5 N NaOH, again centrifuged, and the absorbance of TGA derivatives in the supernatant measured at 280 nm. Results were expressed as the increase in *A*_280 nm_/g AIR fresh weight (FW).

#### 2.5.3. PAL Activity Assay in Tomato Roots

Enzyme extraction steps were carried out at 4°C. 1 g fresh weight of root tissue was crushed in liquid nitrogen in presence of 20% (w/w) polyvinyl pyrrolidone (PVP) and then extracted with 5 mL of 100 mM homogenization buffer (Tris-HCl, pH 8.0). The suspension was homogenized for 1 min and then centrifuged at 10 000 ×g for 30 min. PAL was assayed directly in the supernatant after concentration through Amicon Ultra-4 CFU membrane (Millipore, Bedford, USA). 200 mM Tris-HCl (pH 7.0) was used as assay buffer. 20 mM L-phenylalanine was used as substrate of the enzyme in the assay. PAL activity was assayed using a modified method of Sainders and McClure [23]. The reaction was carried out for 60 min at 37°C and the increase in *A*_290 nm_ was recorded at every 15 min interval. The rate of formation *t*-cinnamic acid was taken as a measure of enzyme activity using an increase in absorbance of 0.01 at *A*_290 nm_ as 3.09 nmol of* t*-cinnamic acid formed. The PAL activity was expressed as nkat/mg protein.

#### 2.5.4. POD Activity Assay in Tomato Roots

Enzyme extraction steps were carried out at 4°C. 1 g fresh weight of root tissue was crushed in liquid nitrogen in presence of 1 g polyvinyl pyrophosphate and then extracted with 5 mL of 200 mM Tris-buffer (pH 8.0). The suspension was homogenized for 1 min and then centrifuged at 20 000 ×g for 20 min. Peroxidase activity was determined from the crude enzyme extract (supernatant) after concentration through Amicon Ultra-4 CFU membrane (Millipore, Bedford, USA) using an assay system consisting of 20 mM guaiacol (0.5 mL), 0.1 mM acetate buffer (pH 5.0) (2.1 mL), 40 mM hydrogen peroxide (H_2_O_2_) (0.2 mL), and the enzyme extract (0.2 mL) with a final volume of 3 mL (modified from Chance and Maehly [24]). Oxidation of guaiacol was measured by the increase in absorbance at 470 nm. One unit of enzyme activity represented the amount of enzyme catalyzing the oxidation of 1 *μ*mol of guaiacol in 1 min.

#### 2.5.5. PPO Activity Assay in Elicited Tomato Roots

Root segments (elicited and control) were homogenized (1 : 2 w/v) in 0.1 M potassium phosphate buffer (ice cold extraction buffer, pH 6.8). The homogenate was centrifuged at 20 000 ×g for 30 min at 4°C. The supernatant was used directly in the enzyme assay. The reaction mixture contained 1 mM catechol in 0.05 M sodium phosphate buffer (pH 6.5) and 500 *μ*L enzyme extract. The reference contained only substrate. PPO activity was determined using catechol as substrate and monitoring the increase in absorbance at 405 nm [25]. The linear portion of the activity curve was used to express enzyme activity (nkat/mg protein). One unit was defined as a change in absorbance of 0.001 under the assay conditions.

#### 2.5.6. CAD Activity Assay in Elicited Tomato Roots

CAD was extracted in 0.1 M Tris-HCl (pH 7.5) containing 15 mM *β*-mercaptoethanol, polyethylene glycol (10% v/v), and 5% polyclar. Root segments were homogenized (1 : 2 w/v) and centrifuged at 20 000 ×g for 20 min at 4°C. The supernatants were directly used in the enzyme assay. The reaction mixture consisted of 2.5 mL 0.1 M Tris-HCl (pH 8.8), 200 *μ*L 0.1 M Tris-HCl (pH 8.8) containing 3 mM coniferyl alcohol, 200 *μ*L 0.1 M Tris-HCl (pH 8.8) containing 6 mM NADP, and 100 *μ*L enzyme extract. CAD activity was measured following the oxidation of the appropriate hydroxycinnamyl alcohol at 30°C [26]. Assays with coniferyl alcohol as substrate were monitored by following the formation coniferaldehyde at 400 nm. CAD activity was expressed as nkat/mg protein.

#### 2.5.7. CAT Activity Assay Elicited Tomato Roots

Catalase activity was assayed by measuring the rate of disappearance of H_2_O_2_ at 240 nm as per the method of Cakmak and Marschner [27]. The reaction mixture (2 mL) consisted of 25 mM phosphate buffer (pH 7.0), 10 mM H_2_O_2_, and 0.2 mL enzyme extract. One unit was defined as a change in absorbance of 0.1 under the conditions of the assay. Enzyme activity was expressed as nkat/mg protein.

### 2.6. Disease Assessment

Assessment of disease severity was done according to Ishikawa et al. [28] with modifications. Four weeks after challenge of tomato plants by *Ralsol*, the disease index (on 1 to 5 scale) on each plant was recorded according to vascular browning and the mean value was calculated as the disease severity. For evaluation of vascular browning, the basal stems were cut and vascular browning was rated on a scale where 1 = no symptoms or vascular browning; 2 = 1–25% vascular browning; 3 = 26–50% vascular browning; 4 = 51–75% vascular browning; 5 = more than 75% vascular browning. Similarly, the disease index as regards wilting was recorded on the same scale.

### 2.7. Statistical Analysis

Growing, inoculation, and sampling of plants were done in three independent experiments with three replicates. Roots of three plants were considered as 1 sample. Collected plant material was randomly divided into three parts and analyzed.

## 3. Results 

### 3.1. Total Phenolic Content Determination in Tomato Roots on a Time Course

**Figure 1:**
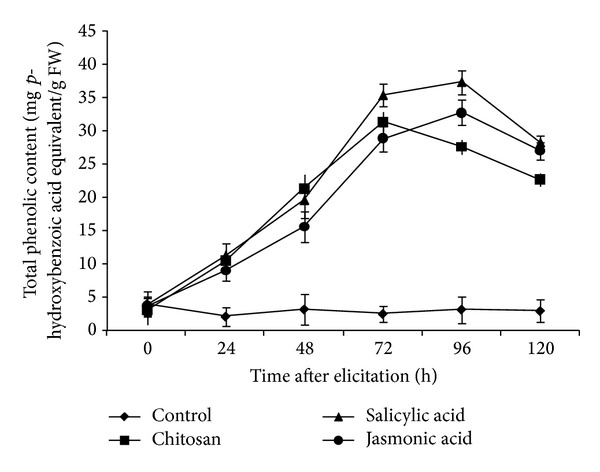
Total phenolic content (expressed as mg 4-hydroxybenzoic acid equivalent/g FW) in tomato roots on a time course. Each value is the mean ± SD from at least three independent extractions.

Total phenolic content in tomato was found to increase starting from 24 h post-elicitation (hpe) under the influence of the elicitors (Figure 1). However, SA was the most effective elicitor that increased total phenolics 12 times compared with control at 96 hpe.

### 3.2. Effect of Elicitors on Lignin in Roots of Tomato

**Figure 2:**
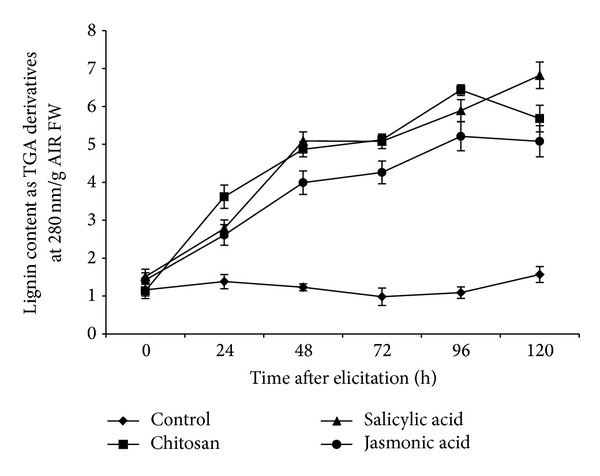
Deposition of lignin (expressed as thioglycolic acid derivatives at 280 nm/g alcohol insoluble residue) in the cell wall on a time course after elicitation of the roots of tomato with different elicitors.

It was observed that all elicitors increased lignin deposition in varying degrees starting from 24 h of elicitation compared to the corresponding control (Figure 2). Most pronounced increase in lignin synthesis was affected by SA followed by CHT. At 120 hpe, highest lignin deposition in the root cell wall was 6.82 TGA derivatives at 280 nm/g AIR FW in case of SA. In case of CHT, the lignin content was highest (5.68 TGA derivatives at 280 nm/g AIR FW) at 96 hpe.

### 3.3. PAL Activity in Elicited Roots of Tomato

**Figure 3:**
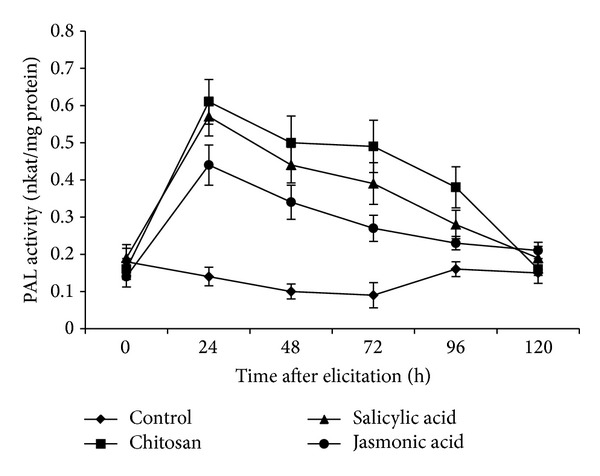
Phenylalanine ammonia lyase (PAL) activity (nkat/mg protein) on a time course after elicitation of the roots of tomato with different elicitors. Values are mean ± SD of triplicate analysis.

Activity of PAL was found to increase sharply, peaking at 24 h of elicitation under the influence of the elicitors (Figure 3). PAL activity was higher in case of CHT. At 24 hpe, the activity of the enzyme in the CHT sample was 4.5 times higher than the control. At the same time point, the activity of the enzyme was more than 4 times higher than control affected by SA. The rise in PAL activity under the influence of elicitors was transient and showed a sharp decreasing trend after 24 h of elicitation of the roots.

### 3.4. POD Activity in Elicited Roots of Tomato

**Figure 4:**
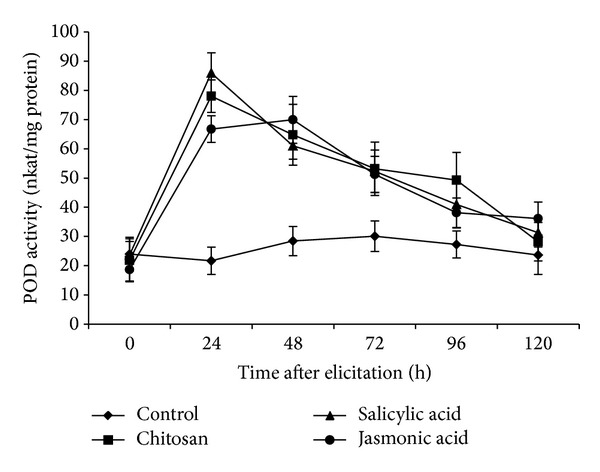
Peroxidase (POD) activity (nkat/mg protein) on a time course after elicitation of the roots of tomato with different elicitors. Values are mean ± SD of triplicate analysis.

Like PAL, POD activity also increased sharply and peak was observed at 24 hpe in case of all three elicitors. However, SA could increase POD activity maximum at 24 hpe followed by CHT and JA. In this elicitation experiment, it was observed that POD activity was about 86 nkat/mg protein at 24 hpe in case of SA. In case of CHT, it was 78 nkat/mg protein at the same time point (Figure 4). POD activity registered a sharp declining trend after 24 hpe in case of these two elicitors, almost reaching the activity level of control plants at 120 hpe. However, JA could sustain the increased level of POD activity up to 48 hpe and then a decrease was observed.

### 3.5. PPO Activity in Elicited Roots of Tomato

**Figure 5:**
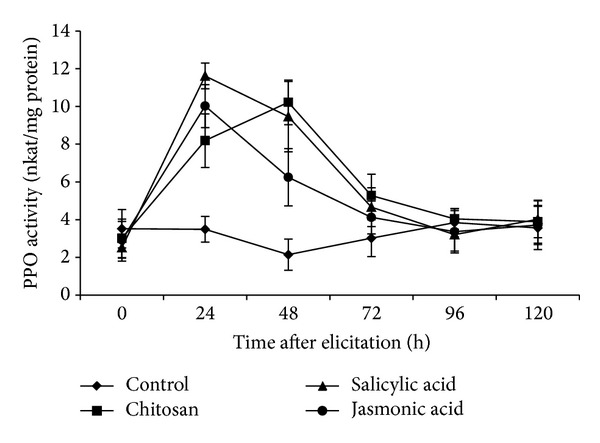
Polyphenol oxidase (PPO) activity (nkat/mg protein) on a time course in elicited and nonelicited (control) roots of tomato. Values are mean ± SD of triplicate analysis.

All the three elicitors could increase PPO activity in varying degrees compared to the control treatments (Figure 5). In case of SA and JA, peak activity of PPO was observed at 24 hpe, whereas maximum activity was at 48 hpe in the case of CHT. After 24 hpe, a rapid decrease of PPO activity was observed for JA. But the decrease in PPO activity was rather gradual in case of the other two elicitors. However, unlike PAL and POD, the activity of PPO returned to the basal level at 96 hpe in case of all elicitors.

### 3.6. CAD Activity in Elicited Roots of Tomato

**Figure 6:**
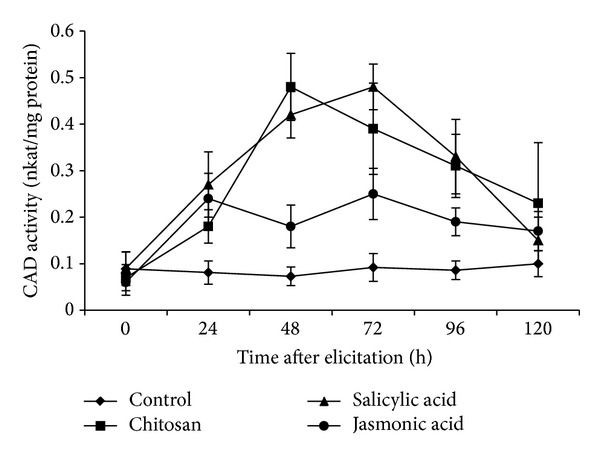
Cinnamyl alcohol dehydrogenase (CAD) activity (nkat/mg protein) on a time course after elicitation of the roots of tomato with different elicitors. Values are mean ± SD of triplicate analysis.

In this elicitation study of defense enzymes, CAD activity showed gradual increase up to 24 h of elicitation. Its activity was observed to reach the highest level (0.49 nkat/mg protein) at 48 h post-elicitation by CHT (Figure 6). CAD activity increased to the maximum (0.48 nkat/mg protein) at 72 hpe under the influence of SA. Activity of the enzyme started falling gradually, but the effect of SA and CHT could maintain a much higher level of CAD activity than the control level even at 120 hpe. JA was not so strong to increase CAD activity in tomato roots.

### 3.7. CAT Activity in Elicited Roots of Tomato

**Figure 7:**
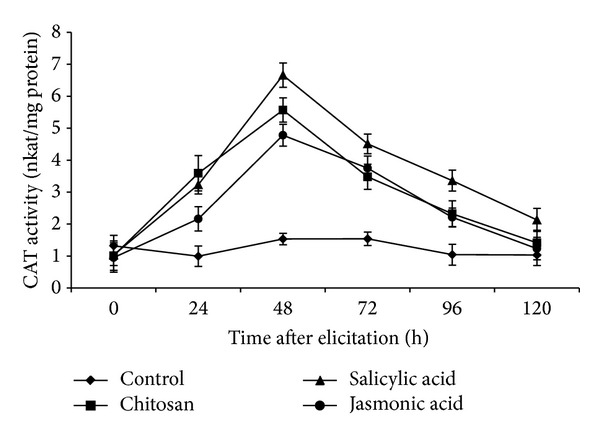
Catalase (CAT) activity (expressed as nkat/mg protein) in roots of tomato plants on a time course after elicitation of the plants with different elicitors. Values are mean ± SD of triplicate analysis.

CAT activity was observed to reach maximum level at 48 hpe in case of all three elicitors. SA elicited the highest CAT activity (6.6 nkat/mg protein) at this time point and from this point onwards, there was a rapid fall in the activity of the enzyme reaching the control level at 120 hpe (Figure 7).

### 3.8. Elicitor Treatment of Tomato Plants Induces Resistance against *Ralsol* Infection

**Figure 8:**
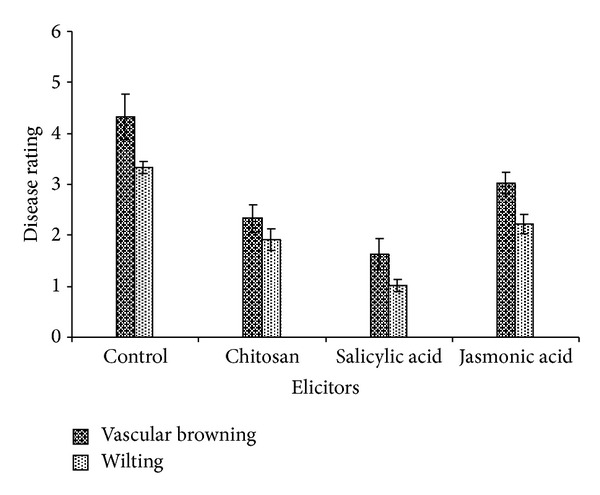
Induction of resistance in tomato plants against *Ralstonia solanacearum* infection by elicitors. Data on vascular browning of roots and leaf yellowing wilting were recorded 4 weeks after challenge of tomato plants by *Ralstonia solanacearum*. Columns represent the mean disease rating on a 1–5 scale as described in Section 2. The experiments were repeated three times with three replicates.

Addition of elicitors to the hydroponics medium significantly affected infection and wilt development by *Ralstonia solanacearum* on tomato plants. The percent of vascular browning and plant wilting was markedly reduced when plants were grown in presence of SA and CHT. Tomato plants inoculated with *Ralstonia solanacearum*, but not receiving SA and CHT treatment, exhibited typical vascular browning and whole plant wilting, while elicitor-treated plants showed less vascular browning and wilting after 4 weeks of the experiment (Figure 8). 

## 4. Discussion

A common feature of induced resistance to disease is the priming of plant tissues by elicitors that allows rapid deployment of active defense mechanisms against invading pathogens. Elicitors are environmentally safe chemicals that can induce effective defense responses in plants. Here we demonstrated increased disease resistance by *Solanum lycopersicum *L. cv. Arka Meghali against bacterial wilt pathogen *Ralstonia solanacearum* upon priming with elicitors and subsequent challenge inoculation of the plant with the pathogen. 

High quantity of antimicrobial phenolic acids was detected in tomato roots as a result of treatment with elicitors, especially SA and CHT. A common host response is the esterification of phenolic acids to host cell wall and crosslinking of such phenylpropanoid esters leads to the formation of lignin-like polymers which provide effective defense against impending pathogen onslaughts. Cell walls may be reinforced through the deposition of lignin, phenolic compounds, suberin, and callose. Lignin blocks pathogen penetration of host cells and inhibits the secretion of virulence effectors, thus contributing to disease resistance [29, 30]. In our study, SA and CHT could induce strong and rapid lignin deposition in tomato root cell wall, which could be specifically targeted against the wilt pathogen* Ralstonia solanacearum*. It has been shown that exogenous SA application prior to inoculation provided increased *Fusarium oxysporum* resistance as evidenced by reduced foliar necrosis and plant death in *Arabidopsis* [31]. Cell wall strengthening, through deposition of lignin preceded by the induction of defense enzymes, played an important role in the defense response of *Lycopersicon esculentum* in reaction to CHT and another elicitor derived from *Fusarium oxysporum* f. sp. *lycopersici*, the causal organism of *Fusarium* wilt of tomato [32]. CHT is one of the most effective members of the oligosaccharin group shown to have plant resistance eliciting function [33]. Besides its antifungal activity, CHT has the potential for inducing defense-related enzymes [34]. CHT treatment induced a significant increase in the activities of PPO and POD and enhanced the content of phenolic compounds in tomato fruits, thus providing protection against gray mould and blue mould diseases [35]. Reports indicate that pretreatment of tomato with an elicitor DL-*β*-aminobutyric acid is effective against *Xanthomonas vesicatoria* [36] and *Clavibacter michiganensis* ssp. *michiganensis* [17].

Increased PAL activity is a key response to pathogen challenge in many plant species and is closely correlated with resistance [37]. PAL regulates secondary metabolism in plants, leading to the biosynthesis of phenylpropanoids as well as the signalling molecule, SA. Enhancement of PAL activities was reported in response to *Rhizoctonia solani* inoculation in cowpea pretreated with SA [38]. PAL activity increased in inoculated leaves of the resistant melon cultivar, resulting in extensive and locally intense deposition of phenolic compounds and lignin surrounding epidermal cells [39]. Addition of 20 *μ*M salicylic acid to *Saussurea medusa *cell cultures resulted in 7.5-fold increase in PAL activity [40]. Similarly, a study has shown that elicitation of grapevine leaves by CHT led marked induction of PAL activity [41]. In our study, the rapid and transient increase in PAL activity in elicited roots is in good agreement with earlier reports. The induction of PAL activity in our study also correlates well with the accumulation of lignin in the cell wall of tomato roots. 

POD is implicated in a variety of functions, such as defense mechanisms [42] and lignification [43]. Nikraftar et al. [44] concluded that POD might be involved in phenolics production in tomato plants, as an effective resistance mechanism in tomato-*Rhizoctonia solani* pathosystem. Spraying of SA on pear plants increased PAL and POD activities greatly and contributed in protection of pear fruits against postharvest diseases [45]. In *Cucurbita pepo* leaves, SA application and zucchini yellow mosaic virus infection could register heightened POD activity [46]. Our study on POD activity indicated that the tomato plants responded actively to SA and CHT elicitation, as has been reported in many earlier cases. We hypothesize a possible involvement of PPO in defense mechanism of tomato roots as manifested by marked increase in the level of PPO activity after elicitation. PPO is a copper-containing enzyme known to be involved in resistance against *R. solanacearum* in resistant tomato cultivars [3]. DL-*β*-aminobutyric acid enhanced PPO activity in tomato plants inoculated with *R. solanacearum* [47]. Besides, overexpression of PPO in transgenic tomato plants enhanced their resistance to *Pseudomonas syringae*, another bacterial pathogen of tomato [48]. Also there are reports that show that PAL and PPO activities increased in resistant tomato cultivars more than those in susceptible and highly susceptible cultivars after inoculation with *Xanthomonas axonopodis* pv. *vesicatoria* [49]. 

In our study, we observed high CAD activity in the roots of tomato during the course of the study. This indicates that the enzyme plays an important role in the lignification process and it correlates very well with the high lignin concentration determined in elicited tomato roots. Higher activity of CAD detected in tomato roots was implicated in cell wall strengthening and defense reaction in response to elicitors, notably CHT [31]. An elicitor made from fungal mycelium extracts induced a rapid stimulation of the monolignol pathway in flax cell suspension cultures, as confirmed by the increase in CAD gene expression [50]. CAT activity was also in higher state in tomato roots treated with the elicitors and subsequently challenged with *R. solanacearum*. CAT is an antioxidative enzyme involved in oxidative burst generated transiently in plant-pathogen interactions. CAT is involved in regulation of H_2_O_2_ levels in plant tissues. Higher concentrations of H_2_O_2_ in resistant than in susceptible tomato cultivars have been reported in tomato-*Ralstonia* interactions [51]. Another study reported that the restriction of *R. solanacearum* growth could be due to the antimicrobial activity of H_2_O_2_, which is strongly increased around bacterial cells, and to the oxidative cross-linking of the cell wall, driven by the rapid accumulation of H_2_O_2_ at the plant cell walls adjacent to attached bacteria [52]. A known plant defense inducer ASM was shown to induce antioxidant enzymes such as CAT, superoxide dismutase, and ascorbate peroxidase, associated with decreased leaf spot severity in tomato [53].

The study by Milling et al. [54] and Chen et al. [2] suggested that tomato fends off the bacterial wilt by multiple defense mechanisms, which involve ET- and SA-related defense signaling pathways. Treatment of plants with SA enhanced local resistance to *B. cinerea* [55]. The plant activator ASM significantly enhanced resistance of tolerant tomato cultivars against bacterial wilt and resulted in yield increase [56]. ASM has been more effective than rhizobacteria in reducing bacterial wilt incidence on susceptible cultivars at low soil populations of *Ralstonia solanacearum* [57]. Silicon induced resistance in tomato against bacterial wilt caused by *Ralstonia solanacearum* [58]. CHT was found capable of inducing resistance in grapevine against anthracnose disease caused by *Sphaceloma ampelinum* [59]. In the present study, we observed reduced vascular browning and wilting symptoms of tomato plants treated with SA and CHT and challenged subsequently with *R. solanacearum*.

## 5. Conclusion

We investigated on important parameters of induced resistance in hydroponically grown tomato (*Solanum lycopersicum*) against* Ralstonia solanacearum* using three elicitors, namely, chitosan, salicylic acid, and jasmonic acid. The results indicate that the elicitors SA and CHT induced effective defense responses in tomato plants against *R. solanacearum*. The reduced disease incidence in tomato by SA and CHT may be a result of cell wall strengthening through deposition of lignin and induction of defense enzymes. It is concluded that SA and CHT may be used as potential resistance inducer against the bacterial pathogen *Ralstonia solanacearum*. The science accumulated on the chemical and physiological functions of CHT associated with plant defense suggests it has a bright future in many aspects of food production [60].

## References

[1] Genin S, Denny TP (2012). Pathogenomics of the *Ralstonia solanacearum* species complex. *Annual Review of Phytopathology*.

[2] Chen Y-Y, Lin Y-M, Chao T-C (2009). Virus-induced gene silencing reveals the involvement of ethylene-, salicylic acid- and mitogen-activated protein kinase-related defense pathways in the resistance of tomato to bacterial wilt. *Physiologia Plantarum*.

[3] Vanitha SC, Niranjana SR, Umesha S (2009). Role of phenylalanine ammonia lyase and polyphenol oxidase in host resistance to bacterial wilt of Tomato. *Journal of Phytopathology*.

[4] Nürnberger T, Brunner F, Kemmerling B, Piater L (2004). Innate immunity in plants and animals: striking similarities and obvious differences. *Immunological Reviews*.

[5] von Röpenack E, Parr A, Schulze-Lefert P (1998). Structural analyses and dynamics of soluble and cell wall-bound phenolics in a broad spectrum resistance to the powdery mildew fungus in barley. *Journal of Biological Chemistry*.

[6] Dixon RA, Achnine L, Kota P, Liu C-J, Reddy MSS, Wang L (2002). The phenylpropanoid pathway and plant defence—a genomics perspective. *Molecular Plant Pathology*.

[7] Fry SC (1987). Intracellular feruloylation of pectic polysaccharides. *Planta*.

[8] dos Santos WD, Ferrarese MDLL, Ferrarese-Filho O (2006). High performance liquid chromatography method for the determination of cinnamyl alcohol dehydrogenase activity in soybean roots. *Plant Physiology and Biochemistry*.

[9] Dardick C, Ronald P (2006). Plant and animal pathogen recognition receptors signal through non-RD kinases. *PLoS Pathogens*.

[10] Kim MG, Da Cunha L, McFall AJ (2005). Two Pseudomonas syringae type III effectors inhibit RIN4-regulated basal defense in Arabidopsis. *Cell*.

[11] Shetty NP, Jørgensen HJL, Jensen JD, Collinge DB, Shetty HS (2008). Roles of reactive oxygen species in interactions between plants and pathogens. *European Journal of Plant Pathology*.

[12] Mandal S, Mallick N, Mitra A (2009). Salicylic acid-induced resistance to *Fusarium oxysporumf. sp. lycopersici* in tomato. *Plant Physiology and Biochemistry*.

[13] Iriti M, Faoro F (2009). Chitosan as a MAMP, searching for a PRR. *Plant Signaling and Behavior*.

[14] Kiirika LM, Stahl F, Wydra K (2013). Phenotypic and molecular characterization of resistance induction by single and combined application of chitosan and silicon in tomato against *Ralstonia solanacearum*. *Physiological and Molecular Plant Pathology*.

[15] Pieterse C, Schaller A, Mauch-Mani B, Conrath U (2006). *Signaling in Plant Resistance Responses: Divergence and Cross-Talk of Defense Pathways*.

[16] Thordal-Christensen H, Zhang Z, Wei Y, Collinge DB (1997). Subcellular localization of H_2_O_2_ in plants. H_2_O_2_ accumulation in papillae and hypersensitive response during the barley-powdery mildew interaction. *Plant Journal*.

[17] Baysal Ö, Gürsoy YZ, Örnek H, Duru A (2005). Induction of oxidants in tomato leaves treated with DL-*β*-Amino butyric acid (BABA) and infected with *Clavibacter michiganensis* ssp. *michiganensis*. *European Journal of Plant Pathology*.

[18] Barilli E, Prats E, Rubiales D (2010). Benzothiadiazole and BABA improve resistance to *Uromyces pisi* (Pers.) Wint. in *Pisum sativum* L. with an enhancement of enzymatic activities and total phenolic content. *European Journal of Plant Pathology*.

[19] Moerschbacher BM, Flott BE, Noll BE, Reisener HJ (1990). On the specificity of an elicitor preparation from stem rust which induces lignification in wheat leaves. *Plant Physiology and Biochemistry*.

[20] Villegus M, Brodelius PE (1990). Elicitor-induced hydroxycinnamoyl-CoA: tyramine hydroxycinnamoyltransferase in plant cell suspension cultures. *Physiologia Plantarum*.

[21] Mandal S, Mitra A, Mallick N (2009). Time course study on accumulation of cell wall-bound phenolics and activities of defense enzymes in tomato roots in relation to *Fusarium* wilt. *World Journal of Microbiology and Biotechnology*.

[22] Bruce RJ, West CA (1989). Elicitation of lignin biosynthesis and isoperoxidase activity by pectic fragments in suspension cultures of castor bean. *Plant Physiology*.

[23] Sainders JA, McClure JW (1975). Phytochrome controlled phenylalanine ammonia lyase in *Hordeum vulgare* plastids. *Phytochemistry*.

[24] Chance B, Maehly M, Colowick SP, Kaplan NP (1995). Assays of catalases and peroxidases. *Methods in Enzymology*.

[25] Gauillard F, Richard-Forget F, Nicolas J (1993). New spectrophotometric assay for polyphenol oxidase activity. *Analytical Biochemistry*.

[26] Wyrambik D, Grisebach H (1975). Purification and properties of isoenzymes of cinnamyl alcohol dehydrogenase from soybean cell suspension cultures. *European Journal of Biochemistry*.

[27] Cakmak I, Marschner H (1992). Magnesium deficiency and high light intensity enhance activities of superoxide dismutase, ascorbate peroxidase, and glutathione reductase in bean leaves. *Plant Physiology*.

[28] Ishikawa R, Shirouzu K, Nakashita H (2005). Foliar spray of validamycin A or validoxylamine A controls tomato *Fusarium* wilt. *Phytopathology*.

[29] Luna E, Pastor V, Robert J, Flors V, Mauch-Mani B, Ton J (2011). Callose deposition: a multifaceted plant defense response. *Molecular Plant-Microbe Interactions*.

[30] Park P, Ikeda K-I (2008). Ultrastructural analysis of responses of host and fungal cells during plant infection. *Journal of General Plant Pathology*.

[31] Edgar CI, McGrath KC, Dombrecht B (2006). Salicylic acid mediates resistance to the vascular wilt pathogen *Fusarium oxysporum* in the model host *Arabidopsis thaliana*. *Australasian Plant Pathology*.

[32] Mandal S, Mitra A (2007). Reinforcement of cell wall in roots of *Lycopersicon esculentum* through induction of phenolic compounds and lignin by elicitors. *Physiological and Molecular Plant Pathology*.

[33] Yin H, Zhao X, Bai X, du Y (2010). Molecular cloning and characterization of a *Brassica napus* L. map kinase involved in oligochitosan-induced defense signaling. *Plant Molecular Biology Reporter*.

[34] Bautista-Baños S, Hernández-Lauzardo AN, Velázquez-Del Valle MG (2006). Chitosan as a potential natural compound to control pre and postharvest diseases of horticultural commodities. *Crop Protection*.

[35] Liu J, Tian S, Meng X, Xu Y (2007). Effects of chitosan on control of postharvest diseases and physiological responses of tomato fruit. *Postharvest Biology and Technology*.

[36] Cohen YR (2002). *β*-aminobutyric acid-induced resistance against plant pathogens. *Plant Disease*.

[37] Pallas JA, Paiva NL, Lamb C, Dixon RA (1996). Tobacco plants epigenetically suppressed in phenylalanine ammonia-lyase expression do not develop systemic acquired resistance in response to infection by tobacco mosaic virus. *Plant Journal*.

[38] Chandra A, Saxena R, Dubey A, Saxena P (2007). Change in phenylalanine ammonia lyase activity and isozyme patterns of polyphenol oxidase and peroxidase by salicylic acid leading to enhance resistance in cowpea against *Rhizoctonia solani*. *Acta Physiologiae Plantarum*.

[39] Ge Y, Bi Y, Guest DI (2013). Defence responses in leaves of resistant and susceptible melon (*Cucumis melo* L.) cultivars infected with *Colletotrichum lagenarium*. *Physiological and Molecular Plant Patholog*.

[40] Yu Z-Z, Fu C-X, Han Y-S, Li Y-X, Zhao D-X (2006). Salicylic acid enhances jaceosidin and syringin production in cell cultures of *Saussurea medusa*. *Biotechnology Letters*.

[41] Trotel-Aziz P, Couderchet M, Vernet G, Aziz A (2006). Chitosan stimulates defense reactions in grapevine leaves and inhibits development of *Botrytis cinerea*. *European Journal of Plant Pathology*.

[42] Bradley DJ, Kjellbom P, Lamb CJ (1992). Elicitor- and wound-induced oxidative cross-linking of a proline-rich plant cell wall protein: a novel, rapid defense response. *Cell*.

[43] Bleea KA, Choib JW, O’Connella AP, Schuchc W, Lewis NG, Bolwell GP (2003). A lignin-specific peroxidase in tobacco whose antisense suppression leads to vascular tissue modification. *Phytochemistry*.

[44] Nikraftar F, Taheri P, Rastegar MF, Tarighi S (2013). Tomato partial resistance to *Rhizoctonia solani* involves antioxidative defense mechanisms. *Physiological and Molecular Plant Patholog*.

[45] Cao J, Zeng K, Jiang W (2006). Enhancement of postharvest disease resistance in *Ya Li* pear (*Pyrus bretschneideri*) fruit by salicylic acid sprays on the trees during fruit growth. *European Journal of Plant Pathology*.

[46] Radwan DEM, Fayez KA, Younis Mahmoud S, Hamad A, Lu G (2007). Physiological and metabolic changes of *Cucurbita pepo* leaves in response to zucchini yellow mosaic virus (ZYMV) infection and salicylic acid treatments. *Plant Physiology and Biochemistry*.

[47] Hassan MAE, Abo-Elyousr KAM (2013). Activation of tomato plant defence responses against bacterial wilt caused by *Ralstonia solanacearum* using DL-3-aminobutyric acid (BABA). *European Journal of Plant Pathology*.

[48] Li L, Steffens JC (2002). Overexpression of polyphenol oxidase in transgenic tomato plants results in enhanced bacterial disease resistance. *Planta*.

[49] Kavitha R, Umesha S (2008). Regulation of defense-related enzymes associated with bacterial spot resistance in tomato. *Phytoparasitica*.

[50] Hano C, Addi M, Bensaddek L (2006). Differential accumulation of monolignol-derived compounds in elicited flax (*Linum usitatissimum*) cell suspension cultures. *Planta*.

[51] Mandal S, Das RK, Mishra S (2011). Differential occurrence of oxidative burst and antioxidative mechanism in compatible and incompatible interactions of tomato and *Ralstonia solanacearum*. *Plant Physiology and Biochemistry*.

[52] Buonaurio R, Barka EA, Clément C (2008). Infection and plant defense responses during plant-bacterial interaction. *Plant-Microbe Interactions*.

[53] Cavalcanti FR, Resende MLV, Carvalho CPS, Silveira JAG, Oliveira JTA (2007). An aqueous suspension of *Crinipellis perniciosa* mycelium activates tomato defence responses against *Xanthomonas vesicatoria*. *Crop Protection*.

[54] Milling A, Babujee L, Allen C (2011). *Ralstonia solanacearum* extracellular polysaccharide is a specific elicitor of defense responses in wilt-resistant tomato plants. *PLoS ONE*.

[55] Ferrari S, Plotnikova JM, De Lorenzo G, Ausubel FM (2003). Arabidopsis local resistance to *Botrytis cinerea* involves salicylic acid and camalexin and requires EDS4 and PAD2, but not SID2, EDS5 or PAD4. *Plant Journal*.

[56] Pradhanang PM, Ji P, Momol MT, Olson SM, Mayfield JL, Jones JB (2005). Application of acibenzolar-S-methyl enhances host resistance in tomato against *Ralstonia solanacearum*. *Plant Disease*.

[57] Anith KN, Momol MT, Kloepper JW, Marois JJ, Olson SM, Jones JB (2004). Efficacy of plant growth-promoting rhizobacteria, acibenzolar-S-methyl, and soil amendment for integrated management of bacterial wilt on tomato. *Plant Disease*.

[58] Ghareeb H, Bozsó Z, Ott PG, Repenning C, Stahl F, Wydra K (2011). Transcriptome of silicon-induced resistance against *Ralstonia solanacearum* in the silicon non-accumulator tomato implicates priming effect. *Physiological and Molecular Plant Pathology*.

[59] Prakongkha I, Sompong M, Wongkaew S, Athinuwat D, Buensanteai N (2013). Changes in salicylic acid in grapevine treated with chitosan and BTH against *Sphaceloma ampelinum*, the causal agent of grapevine anthracnose. *African Journal of Microbiology Research*.

[60] Hadwiger LA (2013). Plant science review: multiple effects of chitosan on plant systems: solid science or hype. *Plant Science*.

